# CRIF1 Deficiency Induces p66shc-Mediated Oxidative Stress and Endothelial Activation

**DOI:** 10.1371/journal.pone.0098670

**Published:** 2014-06-06

**Authors:** Harsha Nagar, Saet-byel Jung, Sun Kwan Kwon, Jung-Bum Park, Minho Shong, Hee-Jung Song, Byeong Hwa Jeon, Kaikobad Irani, Cuk-Seong Kim

**Affiliations:** 1 Department of physiology, School of Medicine, Chungnam National University, Daejeon, Republic of Korea; 2 Department of Endocrinology, Chungnam National University Hospital, Daejeon, Republic of Korea; 3 Department of neurology, Chungnam National University Hospital, Daejeon, Republic of Korea; 4 Division of Cardiovascular Medicine, Department of Internal Medicine, University of Iowa Carver College of Medicine, Iowa City, Iowa, United States of America; University Hospital Medical Centre, Germany

## Abstract

Mitochondrial dysfunction has been implicated in the pathophysiology of various cardiovascular diseases. CRIF1 is a protein present in the mitochondria associated with large mitoribosomal subunits, and CRIF1 knockdown induces mitochondrial dysfunction and promotes ROS production. p66shc is a redox enzyme implicated in mitochondrial ROS generation and translation of oxidative signals and, therefore, is a key factor for oxidative stress in endothelial cells. In this study, we investigated whether mitochondrial dysfunction induced by CRIF1 knockdown induces p66shc stimulation and plays any role in mitochondrial dysfunction-induced endothelial activation. Knockdown of CRIF1 decreased the expression of mitochondrial oxidative phosphorylation (OXPHOS) complexes I, III and IV, leading to increased mitochondrial ROS (mtROS) and hyperpolarization of the mitochondrial membrane potential. Knockdown of CRIF1 also stimulated phosphorylation of p66shc and increased cytosolic ROS in endothelial cells. Furthermore, the expression of vascular cell adhesion molecule-1 and endoplasmic reticulum stress proteins were increased upon CRIF1 knockdown in endothelial cells. However, p66shc knockdown blunted the alteration in mitochondrial dynamics and ROS production in CRIF1 knockdown endothelial cells. In addition, p66shc knockdown reduced the CRIF1 knockdown-induced increases in adhesion between monocytes and endothelial cells. Taken together, these results suggest that CRIF1 knockdown partially induces endothelial activation via increased ROS production and phosphorylation of p66shc.

## Introduction

Oxidative stress has a negative impact on vascular homeostasis by controlling a number of signaling pathways relevant to cardiovascular disease. Oxidative stress results from an imbalance between reactive oxygen species (ROS) generation and removal. In normal cells, ROS formation regulates diverse signaling pathways, but in cardiovascular diseases, ROS overwhelm antioxidant defenses, causing damage [1].

Mitochondria are highly dynamic organelles in eukaryotic cells that play a crucial role in vascular pathophysiology [2]. Mitochondria are a potential target of classical risk factors for cardiovascular disease, such as diabetes, oxidized low-density lipoprotein (oxLDL), aging, smoking, and shear stress [3]. Moreover, mitochondrial damage affects endothelial function [2] in parallel with an increase in ROS [4] and progression of atherosclerosis [5]. A number of different ROS production sites have been identified within mitochondria, the most active of which are complex I and complex III of the oxidative phosphorylation (OXPHOS) chain [6], [7]. OXPHOS is composed of five multiple subunit complexes embedded in the inner mitochondrial membrane. Electrons are transferred from NADH to molecular oxygen through an electron transport chain (ETC), which consists of complexes I (NADH dehydrogenase), II (succinate-ubiquinoneoxidoreductase), III (ubiquinol-cytochromeoxidoreductase), and IV (cytochrome *c* oxidase). Complex V (ATP synthase) condenses ADP and inorganic phosphate to synthesize ATP [8]. Even though mitochondria may not be required for endothelial ATP generation, mitochondrial activity is important for modulating signaling via ROS. In most cells, mitochondrial respiration accounts for a substantial proportion of the ROS produced [9], and in endothelial cells, ROS are produced as a by-product of mitochondrial respiration [10]. Thus, a general principle appears to be emerging in which mitochondrial ROS signal to and trigger ROS production from other cellular sources [11].

CRIF1 is present in the mitochondria and is associated with large mitoribosomal subunits, located near the polypeptide exit tunnel. Deficiency of CRIF1 leads to various defects, such as aberrant synthesis and defective insertion of mtDNA-encoded nascent OXPHOS polypeptides into the inner membrane of the mitochondria [12]. Deficiency of CRIF1 also causes deterioration of mitochondrial function resulting from loss of assembly in OXPHOS complexes I, III and IV, as well as considerably low levels of basal and carbonyl cyanide *m*-chloro phenyl hydrazine (CCCP)-stimulated mitochondrial oxygen consumption and high levels of mitochondrial ROS [13].

p66shc is a ubiquitously expressed protein belonging to the shcA family of adaptor proteins. The mammalian Shc adaptor protein has three isoforms of 46, 52, and 66 kDa (p46shc, p52shc, and p66shc). p66shc is the only isoform that acts as a redox enzyme and has been implicated in mitochondrial ROS generation and translation of oxidative signals [14]. In the vasculature, p66shc plays an important role in endothelial dysfunction associated with pathophysiological conditions, such as atherosclerosis [15]. In response to stimulation by various growth factors and cytokines, the shcA proteins are phosphorylated on tyrosine residues [16]–[18]. However, p66shc is also primarily phosphorylated on ser36 after exposure to oxidative stress from H_2_O_2_ or ultraviolet light [19]–[22]. Phosphorylation on ser36 is essential for imparting increased susceptibility to oxidative stress and is critical for the cell death response evoked by oxidative damage [23].

In this study, we hypothesized that mitochondrial dysfunction induced by CRIF1 deficiency stimulates ROS production, leads to endothelial activation and partially mediates endothelial activation by p66shc.

## Materials and Methods

### Cell culture and transfection

The Animal Care Committee of Chungnam National University approved the animal care and all experimental procedures conducted in this study. Mouse MS-1 endothelial cells were purchased from the American Type Culture Collection (Manassas, VA, USA) and cultured in Dulbecco's Modified Eagle Medium with 5% fetal bovine serum, 10 U/ml penicillin, and 10 µg/ml streptomycin. Human umbilical vein endothelial cells (HUVECs) were purchased from Clonetics (San Diego, CA, USA) and cultured in Endothelial Growth Medium-2 from Lonza (Walkersville, MD). Sub-confluent, proliferating HUVECs were used at passages 2–8. U937 monocytes were purchased from Clonetics and cultured in RPMI 1640 media from Walgene Inc. (Daegu, South Korea). MS-1 cells and HUVECs were transfected with short interfering RNA (siRNA) for *Crif1* (mouse siRNA sequence: sense-5′-AAUAGUUCCUGGAAGCGAGCACUCC-3′ and antisense-5′-GGAGUGCUCGCUUCCAGGAACUAUU-3′ and human siRNA sequence: sense-5′-UGGAGGCCGAAGAACGCGAAUGGUA-3′ and antisense-5′-UACCAUUCGCGUUCUUCGGCCUCCA-3′) and negative control siRNA using Lipofactamine 2000 reagent from Invitrogen (Carlsbad, CA, USA) per the manufacturer's recommendations.

### Antibodies and Western blotting

Rabbit polyclonal Anti-Crif1, rabbit polyclonal anti-vascular cell adhesion molecule-1 (VCAM-1) antibodies were purchased from Santa Cruz Biotechnology (CA, USA). Mouse monoclonal C/EBP homology protein (CHOP), rabbit monoclonal eukaryotic initiation factor 2 (eIF2)-α antibodies were purchased from Cell signaling (USA). Mouse monoclonal antibodies against OXPHOS complex subunits (NDUFA9, SDHA, UQCRC2, COX4 and ATP5A1) were purchased from Invitrogen, mouse polyclonal anti-phospho-ser36-p66shc antibody from Calbiochem (CA, USA) and rabbit polyclonal anti-Shc antibody from BD Biosciences (NJ, USA). Western blotting of 30 µg whole-cell lysates was performed using appropriate primary and secondary antibodies. Blots were imaged using a chemiluminescence assay kit from Pharmacia-Amersham (Freiburg, Germany), and band densities were quantified using a Gel Doc 2000 Chemi Doc system and the Quantity One software from Bio-Rad (Hercules, CA). Values were normalized to a β-actin loading control.

### Measurement of mitochondrial ROS

The levels of mitochondrial ROS in endothelial cells were measured by MitoSOX red from Invitrogen. After 48 h of transfection with control or Crif1 siRNA, cells were washed twice with PBS, trypsinized, and the cell suspension incubated in complete medium with 3 µM MitoSOX at 37°C for 15 min in the dark. During the incubation period, each sample was agitated every 5 min to ensure that the reagent reacted sufficiently with the ROS. To reduce the fluorescence background, each sample was washed twice with PBS before detecting the fluorescence intensity of mitoSOX red using the Fluoroskan Ascent fluorescence reader (Thermo Scientific) at an excitation and emission of 530 nm and 590 nm, respectively.

### Measurement of mitochondrial membrane potential

Changes in mitochondrial membrane potential (MMP) after transfection with control or Crif1 siRNA were measured using tetramethylrhodamine (TMRE) dye. TMRE is a membrane potential sensitive dye which translocates into mitochondria. The fluorescence intensity of TMRE is directly proportional to the mitochondrial membrane potential. After 48 h of transfection, cells were washed twice with PBS, trypsinized and the cell suspension collected. TMRE (100 nM) was added to each sample, and the cells were incubated in complete medium at 37°C for 15 min in the dark. During the incubation period, each sample was agitated every 5 min. To reduce the fluorescence background, each sample was washed twice with PBS before detecting the fluorescence intensity using the Fluoroskan Ascent fluorescence reader at an excitation and emission of 530 nm and 590 nm, respectively.

### Hydrogen peroxide measurement

The H_2_O_2_ level of endothelial cells was measured using the Amplex Red Hydrogen Peroxide Assay Kit from Molecular Probes (Invitrogen) according to the manufacturer's instructions. Briefly, the reaction mixture contained 50 µM Amplex Red and 0.1 U/ml HRP in Krebs-Ringer phosphate (KRPG) buffer (145 mM NaCl, 5.7 mM sodium phosphate, 4.86 mM KCl, 0.54 mM CaCl_2_, 1.22 mM MgSO_4_, 5.5 mM glucose, pH 7.35). Each reaction volume was 100 µl, and 1.5×10^4^ cells suspended in a 20-µl volume of KRPG buffer were added to the 100-µl reaction mixture. For the negative control, 20 µl KRPG buffer without cells was added to a separate 100-µl volume of reaction mixture. Amplex Red is specifically sensitive to H_2_O_2_. Oxidation of Amplex Red by H_2_O_2_ in the presence of HRP produces highly fluorescent resorufin. The level of resorufin produced was quantified using the Fluoroskan Ascent at an excitation and emission of 530 nm and 590 nm, respectively.

### Oxygen consumption rate (OCR)

OCR was measured using a Seahorse XF-24 analyzer (Seahorse Bioscience). The day before OCR measurement, sensor cartridge was calibrated with calibration buffer (Seahorse Bioscience) at 37°C. After 48 hours of transfection with control or Crif1 siRNA, cells were washed twice with XF assay media without sodium bicarbonate and phenol red and incubated in a 37°C incubator for 45 min upto 1 hour before calibration. Three readings were taken after addition of each mitochondrial inhibitor before injection of the subsequent inhibitors. The mitochondrial inhibitors used were oligomycin (2 µg/ml), carbonyl cyanide m-chlorophenyl hydrazine (CCCP, 10 µM) and rotenone (2 µM). OCR was automatically calculated and recorded by the sensor cartridge and Seahorse XF-24 software. The plates were saved and the protein concentration was calculated to confirm that there were an approximately equal number of cells in each well.

### Real-time polymerase-chain reaction

Total RNA from cells was isolated using the acid guanidinium thiocyanate–phenol–chloroform method. Complementary DNA (cDNA) was prepared from total RNA using MAXIME RT Premix kit (iNTRON biotechnology, South Korea). Real-time polymerase chain reaction (PCR) was performed using the Prism 7000 Sequence Detection System (Applied Biosystems, Foster City, CA, USA) with the Super Script III Platinum SYBR Green One-Step qRT-PCR Kit (Invitrogen, Carlsbad, CA, USA). Primers for mouse VCAM-1 were as follows: sense-5′-TGAACCCAAACAGAGGCAGAG-3′ and antisense-5′-GGTATCCCATCACTTGAGCAG-3′. Mouse glyceraldehyde 3-phosphate dehydrogenase (GAPDH) was used as an internal control. Primers for mouse GAPDH were as follows: sense- 5′-ATGACATCAAGAAGGTGGTG-3′ and antisense-5′-CATACCAGG AAAATGAGCTTG-3′. Dissociation curves were monitored to check the aberrant formation of primer-dimers. Results were interpreted by the relative quantity method (ΔΔCt).

### Adenoviral infection

Recombinant adenovirus encoding shRNA for p66shc (Adp66shcRNAi) was obtained from Dr. Kaikobad Irani (University of Pittsburgh). The recombinant adenovirus encodes a short hairpin loop RNA with a 19-mer sequence corresponding to bases 45–63 of p66shc cDNA. These nucleotides are in the 330-bp coding region of the N-terminal CH_2_ domain and are unique to p66shc mRNA. Adp66shc was generated using the AdEasy system as described previously in [24]. MS-1 cells were infected with the adenovirus at a multiplicity of infection (MOI) ranging from 10–1,000 in 6-well tissue culture plates. After 24 h of infection, the protein expression of total p66shc was determined by Western blot analysis. Thereafter, the adenovirus was used at 100 MOI for the subsequent experiments. Adenovirus β-galactosidase (Adβ-gal), encoding the inert LacZ gene, was used as a control virus for the experiments.

### Cell adhesion assay

HUVECs were infected with Adp66shcRNAi or AdLacZ (as a control) for 24 h and transfected with Crif1 siRNA for 48 h as described previously. U937 cells were incubated with the HUVECs for 30 min at 37°C in the dark. Monocyte adhesion was quantified by counting the cells. Wells containing HUVEC without U937s were used as blanks.

### Statistical analysis

All experiments were performed at least three times. Data are presented as the means ± SEM. Statistical significance was determined using the Student's *t*-test and by a *p*-value<0.05.

## Results

### CRIF1 deficiency impaired mitochondrial OXPHOS function in endothelial cells

**Figure 1:**
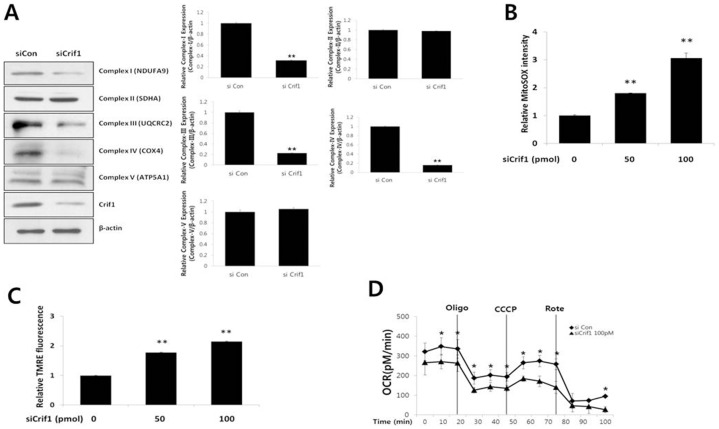
Loss of OXPHOS complexes and mitochondrial dysfunction in *Crif1*-silenced cells. (A) CRIF1 deficiency decreased OXPHOS complex subunit I, III and IV OXPHOS complex subunits, as determined by Western blotting using the appropriate antibodies. β-actin is shown as a loading control. OXPHOS complexes protein expression levels were quantified by densitometric analysis (right panel). Western blots are representative of three independent experiments. (B) CRIF1 deficiency increased mitochondrial ROS. Relative fluorescence of MitoSOX red was used as a measure of mitochondrial ROS levels (C) CRIF1 deficiency increased the mitochondrial membrane potential. Relative fluorescence of TMRE was used as a measure of mitochondrial membrane potential. (D) Oxygen consumption rates (OCR) were measured using a Seahorse XF-24 flux analyzer. Oligo: Oligomycin, CCCP: Carbonyl cyanide *m*-chloro phenyl hydrazine, Rote: Rotenone. Data represent means ± SEM of three independent experiments, *P<0.05, ** P<0.01, compared with the control.

CRIF1 is localized in the mitochondria of mouse embryonic fibroblasts, and mitochondrial OXPHOS is impaired in brain-specific *Crif1* knockout mice [13]. Therefore, we first examined the role of CRIF1 on mitochondrial function in endothelial cells. To silence the expression of CRIF1 protein in cultured endothelial cells, MS-1 cells were transfected with *Crif1* siRNA. As shown in Figure S1A, CRIF1 expression was markedly reduced by 50 and 100 pmol *Crif1* siRNA compared with the control siRNA 48 h after transfection. For the time-dependent experiments, we used 100 pmol of *Crif1* siRNA over an incubation period of 72 h. As shown in Figure S1, the most effective knockdown of CRIF1 protein was seen between 24 and 48 h of incubation after transfection. Based on these experiments, we chose a concentration of 100 pmol of *Crif1* siRNA and an incubation period of 48 h to achieve effective knockdown of CRIF1 protein for the subsequent experiments. In the next set of experiments, we determined whether silencing *Crif1* down-regulates the protein expression of OXPHOS complex subunits in MS-1 cells. The expression levels of NDUFA9 (Complex I), UQCRC2 (Complex III) and COX4 (Complex IV) were significantly inhibited by CRIF1 deficiency as compared with that induced by the control siRNA (Figure 1A), while expression levels of other OXPHOS complexes showed no change.

To explore the effects of CRIF1 deficiency on mitochondrial OXPHOS function, we next examined mitochondrial ROS production and MMP in MS-1 cells. As shown in Figure 1B, there was a marked increase in MitoSox fluorescence intensity with 100 pmol *Crif1* siRNA compared with the control siRNA 48 h after transfection. This observed change in mitochondrial ROS production was accompanied by a hyperpolarization of the MMP in *Crif1*-silenced cells as compared with the control cells as indicated by an increased relative TMRE fluorescence (Figure 1C). TMRE becomes accumulated following the electric potential across the inner mitochondrial membrane (IMM). Furthermore, the oxygen consumption rates (OCR) were markedly decreased in *Crif1*-silenced cells (Figure 1D). Cells were exposed first to Oligomycin, which inhibits the ATP synthase and caused the expected decrease in OCR in both control and Crif1-silenced MS-1 cells. To measure maximal mitochondrial respiratory capacity, we then treated cells with (CCCP), an uncoupler of mitochondrial respiration. Cells normally increase OCR in response to CCCP to maintain proton gradients and mitochondrial function. Crif1 silenced cells exhibited less of an increase in OCR after CCCP treatment compared to control cells, suggesting that these cells have less spare respiratory capacity than control cells. Administration of Rotenone, a complex I inhibitor, also inhibited the OCR. These data suggest that CRIF1 deficiency results in the loss of mitochondrial OXPHOS function in endothelial cells.

### p66shc mediates CRIF1 deficiency-induced ROS in endothelial cells

**Figure 2:**
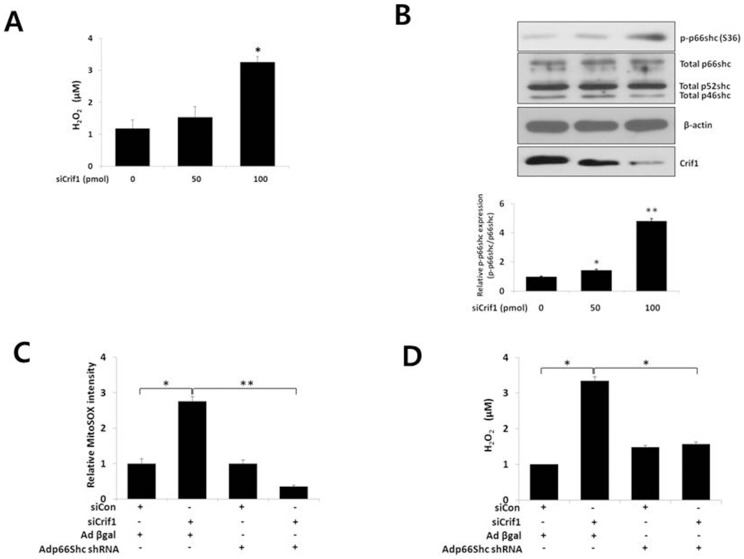
p66shc mediates CRIF1 deficiency-induced mitochondrial ROS and cytosolic ROS production in *Crif1*-silenced cells. MS-1 cells were transfected with *Crif1* siRNA for 48 h. (A) CRIF1 deficiency increased cytosolic ROS, measured using the Amplex Red H_2_O_2_ assay kit. Relative fluorescence of Amplex Red was used as a measure of the H_2_O_2_ released from the cells. (B) CRIF1 deficiency increased phosphorylation of p66shc (p-p66shc) on ser36. p-p66shc levels were measured by Western blotting, which are representative of three independent experiments, and quantified by densitometric analysis (B, lower panel). (C) and (D) p66shc mediated CRIF1 deficiency-induced ROS production. MS-1 cells were infected with Adp66shcRNAi or AdLacZ for 24 h, followed by transfection with *Crif1* siRNA for 48 h. Data represent means ± SEM of three independent experiments, *P<0.05, ** P<0.01 compared with the control.

Increased mitochondrial ROS stimulates NADPH oxidase activation, leading to an increase in the cytosolic ROS levels in MS-1 cells [4]. p66shc is a key protein in the regulation of oxidative stress. Thus, we examined the role of CRIF1 deficiency on ROS production in MS-1 cells. Overall, cellular ROS generation was augmented in *Crif1*-silenced cells compared with control cells in a concentration-dependent manner (Figure 2A). When we assessed whether p66shc mediates CRIF1 deficiency-induced ROS production in endothelial cells, we found that CRIF1 deficiency increased the phosphorylation of p66shc at ser36, but no changes in total protein levels of p66shc were noted (Figure 2B). However, p66shc knockdown using an adenovirus encoding shRNA for p66shc (Adp66shcRNAi) prevented CRIF1 deficiency-induced increases in mitochondrial ROS levels, as well as cytosolic ROS levels in MS-1 cells (Figure 2C and D). These data suggest that p66shc plays an important role in CRIF1 deficiency-induced oxidative stress in endothelial cells

### CRIF1 deficiency induces stimulation of ER stress response in MS-1 cells

**Figure 3:**
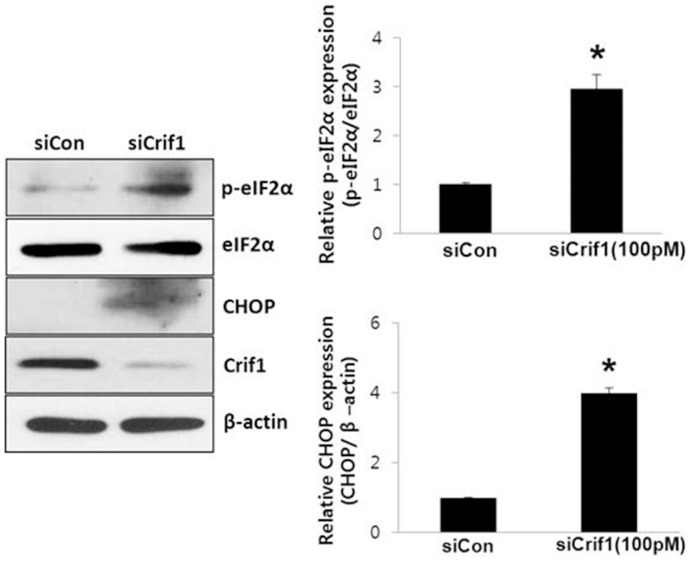
Phosphorylation of eIF2α (p-eIF2α) and CHOP protein expression were up-regulated in *Crif1*-silenced cells. CRIF1 deficiency induced p-eIF2α and CHOP protein expression. p-eIF2α and CHOP protein expression levels were measured by Western blotting. p-eIF2α and CHOP protein expression levels were quantified by densitometric analysis (left panels). All Western blots are representative of three independent experiments, and data are presented as means ± SEM of three independent experiments, *P<0.05 compared with the control.

Intracellular free radicals, such as ROS, act as a major mediator for the cross-talk between ER stress and oxidative stress. ROS stimulates ER stress to activate the unfolded protein response (UPR), which induces an inflammatory response [25]. Because CRIF1 deficiency increased ROS production, we investigated whether CRIF1 deficiency stimulates ER stress in endothelial cells. CRIF1 deficiency significantly stimulated eIF2-α phosphorylation and CHOP expression levels in these cells (Figure 3).

### p66shc mediates CRIF1 deficiency-induced VCAM-1 in MS-1 cells

**Figure 4:**
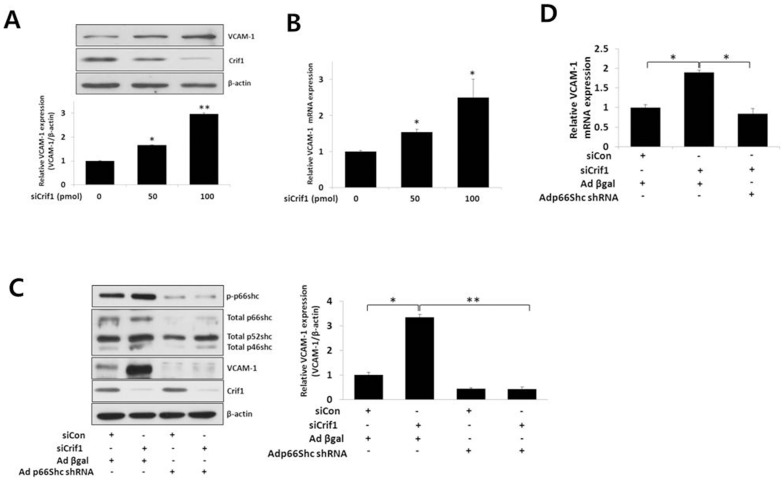
p66shc mediates CRIF1 deficiency-induced VCAM-1 expression in *Crif1* silenced cells. (A) and (B) CRIF1 deficiency increased VCAM-1 protein and mRNA expression levels, respectively. MS-1 cells were transfected with *Crif1* siRNA for 48 h. Protein expression was measured by Western blotting. VCAM-1 protein expression levels were quantified by densitometric analysis (A, lower panel). (C) and (D) p66shc mediates CRIF1 deficiency-induced VCAM-1 protein and mRNA expression levels, respectively. MS-1 cells were infected with Adp66shcRNAi or AdLacZ for 24 h, followed by transfection with *Crif1* siRNA for 48 h. VCAM-1 protein expression levels were quantified by densitometric analysis (C, right panel). All Western blots are representative of three independent experiments and data are presented as means ± SEM of three independent experiments, **P<0.01, *** P<0.001 compared with the control.

ROS mediates up-regulation of inflammatory proteins, such as VCAM-1, in the endothelium and induces vascular activation [26]. Therefore, we examined if CRIF1 deficiency induced any changes in VCAM-1 expression. CRIF1 deficiency increased VCAM-1 protein and mRNA expression in a concentration dependent manner in endothelial cells (Figure 4A and B). Next, we determined whether p66shc mediates the CRIF1 deficiency-induced increase in VCAM-1 expression. The CRIF1 deficiency-induced increase of VCAM-1 expression was blunted significantly by knockdown of p66shc expression, using Adp66shcRNAi, in MS-1 cells (Figure 4C and D).

### p66shc mediates CRIF1 deficiency-induced adhesion of monocytes to HUVECs

**Figure 5:**
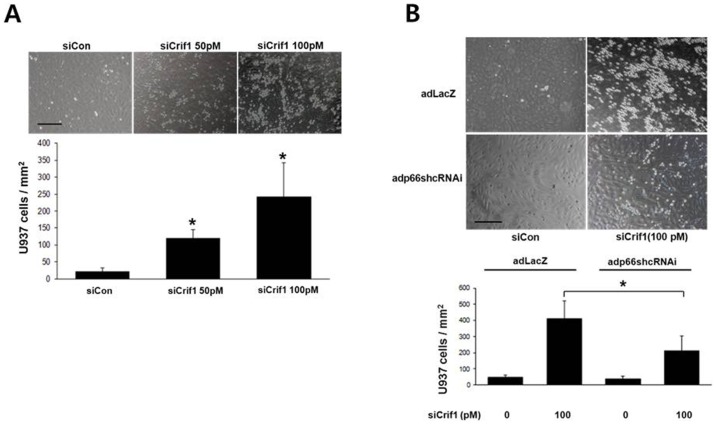
p66shc mediates CRIF1 deficiency-induced adhesion of monocytes to endothelial cells (HUVECs). (A) CRIF1 deficiency induced monocyte adhesion to HUVECs. HUVECs were transfected with *Crif1* siRNA for 48 h. Representative photomicrographs of U937 cells adherent to HUVECs (A, upper panel) and quantification of adherent cells (A, lower panel) are shown. (B) p66shc mediates CRIF1 deficiency-induced adhesion of monocytes to HUVECs. HUVECs infected with Adp66shcRNAi or AdLacZ were transfected with *Crif1* siRNA for 48 h and incubated with U937 cells for 30 min. Representative photomicrographs of U937 cells adherent to HUVECs (B, upper panel) and quantification of adherent cells (B, lower panel) are shown. Data represent means ± SEM of three independent experiments, *P<0.05 compared with the control.

The expression of adhesion molecules such as VCAM-1 in endothelial cells leads to the recruitment of inflammatory leukocytes. Adhesion of these leukocytes to the vascular endothelium depends on the up-regulation of adhesion molecules on endothelial cells. Therefore, we examined whether p66shc is responsible for CRIF1 deficiency-induced adhesion of monocytes to endothelial cells. We performed a cell adhesion assay to investigate the adhesion of monocytic U937 cells to HUVECs following CRIF1 or p66shc knockdown in *Crif1*-silenced HUVECs. As shown in Figure 5A, CRIF1 deficiency stimulated the adhesion of U937 cells to HUVECs. Furthermore, this increase in monocyte adhesion was significantly prevented by knockdown of p66shc in *Crif1*-silenced cells, suggesting that p66shc mediates this adhesion. (Figure 5B).

## Discussion

CRIF1, a CR6/gadd45-interacting protein localized primarily in the mitochondria [13], interacts with the protein components of mitochondrial ribosomes. It is also involved specifically in the regulation of the translation and insertion of mitochondrial OXPHOS complexes. A deficiency in CRIF1 caused a drastic decrease in OXPHOS complexes and increase in mitochondrial ROS production, as well as changes in the mitochondrial membrane potential, indicative of dysfunctional mitochondria. Interestingly, OXPHOS complexes I, III, and IV were decreased in brain-specific *Crif1* knockout mice [13], and complexes I, III, IV and V were decreased in adipose-derived stem cells that lacked *Crif1* [27]. In this study, OXPHOS complexes I, III and IV were decreased in *Crif1*-deficient MS-1 cells (Figure 1A). Thus, the regulation of OXPHOS complexes by CRIF1 deficiency may depend on the cell type.

ROS are constantly produced in the mitochondria as a byproduct of oxidative phosphorylation and play a physiologically important role in the regulation of cell signaling. Thus, mitochondria not only contribute to ATP generation but also help regulate endothelial cell homeostasis through the regulation of ROS. As compared with other cells, endothelial cells depend highly on glycolytic metabolism. However, even though OXPHOS subunits of the mitochondria play a limited role in energy production, they are a major source of ROS production and thus may play a physiologically significant role. Recent studies have shown that increased mitochondrial ROS formation and oxidative mitochondrial DNA damage are related to endothelial dysfunction [4]. In contrast to the rapidly growing knowledge about the relationship between mitochondrial oxidative stress and endothelial dysfunction in endothelial cells, relatively little is known about how mitochondrial dysfunction causes endothelial activation and vascular inflammation. Our work identifies CRIF1 as a novel element important for maintaining mitochondrial function in endothelial cells.

Previous studies have revealed the crucial role that p66shc plays in ROS production within mitochondria and its involvement in cardiovascular diseases [28], [29]. There is also evidence that p66shc is very important for regulating intracellular redox balance and oxidative stress levels and that there is a reduction in intracellular free radicals in cells lacking the p66shc gene [30]. After serine phosphorylation, p66shc moves from the cytosol to the mitochondrial inter-membrane space where it induces mitochondrial H_2_O_2_ production, further increasing intracellular H_2_O_2_ levels [31]. This way it can maintain or increase its own activation via a self-triggered control loop [32], [33]. Therefore, it is now widely accepted that p66shc controls ROS production and oxidative stress leading to mitochondrial and endothelial cell dysfunction. However, the role of p66shc in mitochondrial dysfunction-induced endothelial dysfunction and vascular inflammation remains to be understood.

In this study, we showed increased phosphorylation of p66shc on the ser36 residue following *Crif1* knockdown (Figure 2B). It has been shown previously that ser36 phosphorylation of p66shc is triggered by oxidative stress-inducing stimuli [19], [34] and that the activity of p66shc is strictly dependent on its phosphorylation on ser36. Our data are also consistent with the fact that increased phosphorylation of p66shc triggers ROS production in the cytosol, leading to a further rise in overall ROS levels (Figure 2A). In addition, p66shc knockdown inhibited the increase in both mitochondrial and cytosolic ROS in MS-1 cells (Figure 2C and D), suggesting that p66shc plays an important role in mitochondrial dysfunction- induced endothelial activation. In addition to inhibiting mitochondrial and cytosolic ROS and the mitochondrial dysfunction-induced ER stress response, p66shc knockdown inhibited VCAM-1 expression and the adhesion of monocytes to endothelial cells. These results suggest that p66shc is a dominant, but not the sole mediator, of endothelial dysfunction triggered by mitochondrial dysfunction.

In conclusion, our data provide novel insights into the relationship between mitochondrial dysfunction and endothelial activation. These findings support that *Crif1* deficiency leads to mitochondrial dysfunction and endothelial activation via increased phosphorylation of p66shc and increased oxidative stress. Thus, it is possible that mitochondrial OXPHOS capacity may be an important factor determining the risk of p66shc-mediated endothelial activation.

## Supporting Information

Figure S1 **Crifl Knockdown using** ***Crif1*** **siRNA.** (A) Transfection of 50 and 100 pmol *Crif1* siRNA for 48 h effectively reduced CRIF1 protein expression in a concentration-dependent manner. (B) *Crif1* siRNA transfection reduced CRIF1 protein expression in a time-dependent manner over a 72-h period. The cells were harvested and subjected to Western blot analysis for CRIF1 (A and B, upper panels). β-actin is shown as a loading control. CRIF1 expression levels were quantified by densitometric analysis (A and B, lower panels). All Western blots are representative of three independent experiments, and data are presented as means ± SEM of three independent experiments, *P<0.05, ** P<0.01, compared with the control.
(TIF)
