# Supplementary figures and images for: CRIF1 Deficiency Induces p66shc-Mediated Oxidative Stress and Endothelial Activation
Source: PLoS One. 2014 Jun 6;9(6):e98670. doi: 10.1371/journal.pone.0098670 (PMC4048193; doi:10.1371/journal.pone.0098670)

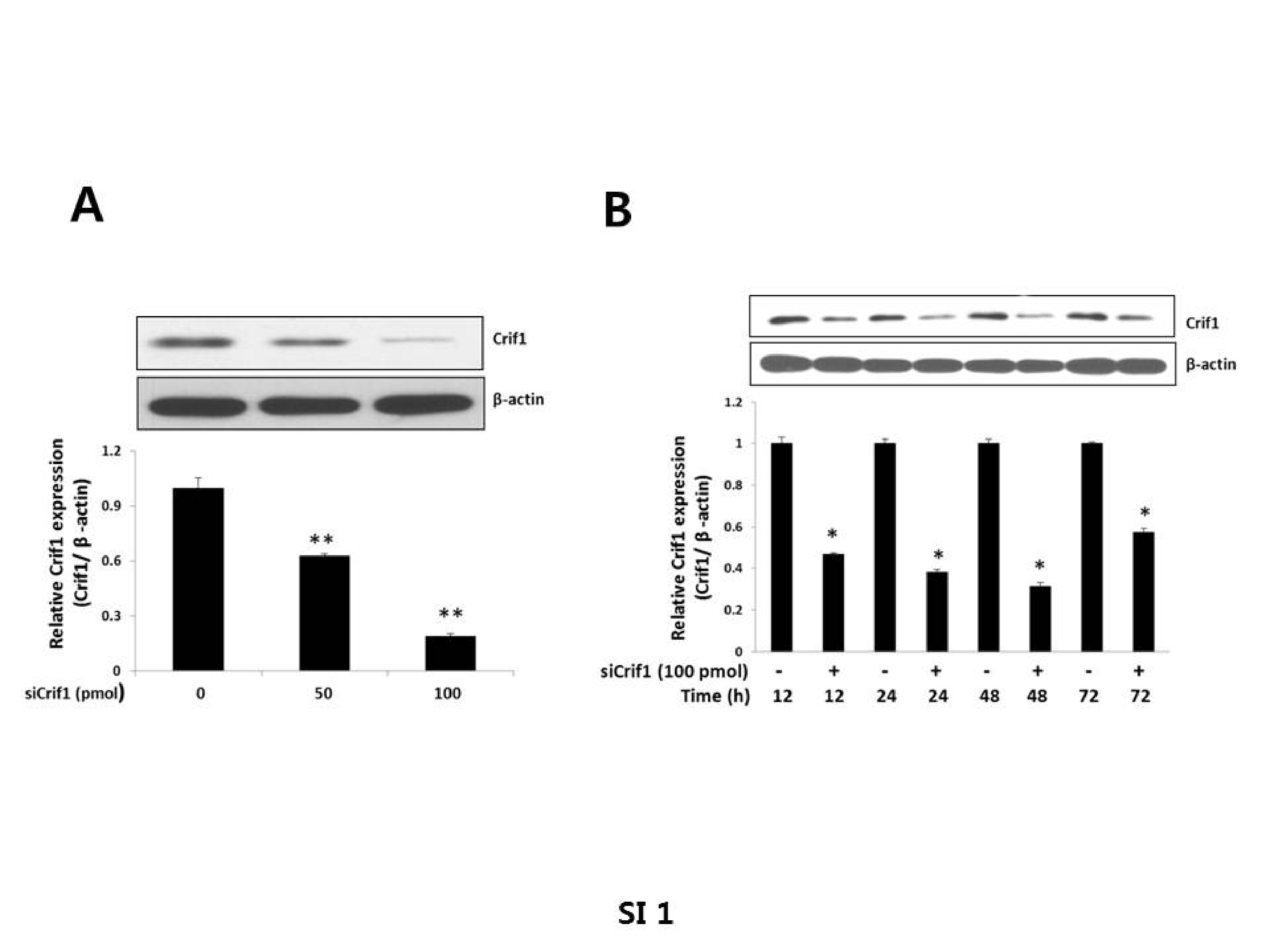

Supplement: Figure S1 — Crifl Knockdown using Crif1 siRNA. (A) Transfection of 50 and 100 pmol Crif1 siRNA for 48 h effectively reduced CRIF1 protein expression in a concentration-dependent manner. (B) Crif1 siRNA transfection reduced CRIF1 protein expression in a time-dependent manner over a 72-h period. The cells were harvested and subjected to Western blot analysis for CRIF1 (A and B, upper panels). β-actin is shown as a loading control. CRIF1 expression levels were quantified by densitometric analysis (A and B, lower panels). All Western blots are representative of three independent experiments, and data are presented as means ± SEM of three independent experiments, *P<0.05, ** P<0.01, compared with the control. (TIF) [file pone.0098670.s001.tif]
